# Dexmedetomidine Attenuates LPS-Stimulated Alveolar Type II Cells' Injury through Upregulation of miR-140-3p and Partial Suppression of PD-L1 Involving Inactivating JNK-Bnip3 Pathway

**DOI:** 10.1155/2022/8433960

**Published:** 2022-07-31

**Authors:** Xianfeng Chen, Juntao Hu, Jie Lai, Zhiyong Zhang, Zhanhong Tang

**Affiliations:** Department of Intensive Care Unit, The First Affiliated Hospital of Guangxi Medical University, Nanning 530021, China

## Abstract

Dexmedetomidine (DEX), which is reported to be a newly discovered, novel *α*-2 adrenoceptor agonist, is known to exhibit anti-inflammatory properties in several diseases. DEX regulates inflammation-related signaling pathways and genes through interactions with several miRNAs. This study verified that expression levels of miR-140-3p were diminished when alveolar type II cells were exposed to LPS. However, the levels of miR-140-3p were confirmed as showing an increase with DEX treatment. These observations revealed that the expression of miR-140-3p was related to the beneficial effects that accompanied the DEX treatment of LPS-induced ALI. In addition, PD-1/PD-L1 expression increased extensively when RLE-6TN cells were induced by LPS. The increased expression was reduced after treatment with DEX. Thus, it appears that the PD-L1 expression was targeted directly by miR-140-3p, resulting in the partial repression of PD-L1 levels, which involved the inhibition of p-JNK and Bnip3 expression. Therefore, DEX was shown to inhibit the PD-L1 expression by promoting partially increased miR-140-3p levels in RLE-6TN cells. DEX also inactivated the JNK-Bnip3 pathway, resulting in the inhibition of inflammation and alleviating alveolar type II cell injury.

## 1. Introduction

The leading type of acute lung injury (ALI) has been reported to be acute respiratory distress syndrome (ARDS). Common causes that lead to ARDS are pulmonary and extrapulmonary pathogenic factors. The primary manifestations of ARDS include increased inflammatory responses in the lungs as well as severe impairment of gas exchange that results from the disruption of the alveolar-capillary barrier leading to pulmonary edema [1, 2]. Inflammation has been reported to be a pervasive and critical component of numerous diseases involving the respiratory airways. It is commonly accepted that abnormal and disrupted inflammatory responses are likely to occur in a range of severe lung diseases, including sepsis, ALI/ARDS, and other vascular diseases [3].

Dexmedetomidine (DEX) has been reported to be a novel, very selective *α*-2 adrenoceptor agonist that demonstrates excellent sedative effects and is frequently used as a sedative in clinical settings. DEX has been shown to reduce tissue injury in rats caused by ischemia-reperfusion by decreasing oxidative stress in the injured tissues and decreasing inflammatory mediator release [4]. For example, DEX can exert a protective effect on LPS-induced ALI, which involved in the regulation of TLR4/NF-*κ*B and PI3K/Akt/mTOR pathways [5]. And, Li et al. reported that DEX can suppress lung apoptosis induced by renal ischemia-reperfusion injury by the regulation of *α*2AR/PI3K/Akt pathway [6].

Recent studies have shown that DEX regulates inflammation-related signaling pathways and genes through interactions with several miRNAs [7]. One miRNA that has been identified is MiR-140, which appears to be associated with the anti-inflammatory effect of DEX. A study in neonatal rats indicated miR-140-5p is actively involved in the protection provided by DEX against hypoxic-ischemic brain injury in the cerebrum [8]. In our study, we found DEX could reverse the decreasing expression of miR-140-3p induced by LPS in cells.

It has been documented that programmed cell death receptor (PD)-1 as well as PD-L, which is a ligand of PD-1, exhibits critical functions as negative regulatory molecules that modulate the activation of leukocytes and maintain antigen tolerance. PD-1 expression has been documented in tissues and organs of the immune system [9]. Furthermore, the expression of PD-L1 occurs on the surface of nonimmune cells as well as immune cells alike, such as endothelial cells associated with blood vessels in the lung and alveolar epithelial cells. Studies have shown that DEX exerts a regulatory effect on PD-1/PD-L1. A rat spinal cord injury model indicated that DEX exposure could induce the upregulation of PD-1, which alleviated neuroinflammation associated with microglial activation [10]. Thus, PD-1 might be involved in the anti-inflammatory effects produced by DEX treatment. However, no reports have been published that document the underlying pathophysiological mechanisms that form the basis by which PD-1/PD-L1 is regulated by DEX in ARDS/ALI.

Therefore, we hypothesis that the molecular mechanisms of DEX inhibited inflammation induced by LPS are involved regulating PD-1/PD-L1 through the actions of miR-140-3p.

## 2. Materials and Methods

### 2.1. Cell Culture

RLE-6TN cells were cultured in the RPMI-1640 medium with 10% fetal bovine serum (FBS, Hyclone) and penicillin-streptomycin (100 U/mL) at 37°C with 5% CO2. DEX was obtained from Abmole. Lipopolysaccharide (LPS) was used to simulate RLE-6TN cells to induce acute lung injury.

### 2.2. CCK-8 Assay of Cell Viability

Cell viability was measured by cell counting kit-8 (CCK-8, Sigma) according to the manufacturer's recommendation. RLE-6TN cells were seeded into 96-well plates at a density of 10000 cells per well overnight at 37°C. Then, cells were divided into 4 groups: blank control group (control), LPS group (20 *μ*g/mL LPS-treated cells for 24 h), DEX group (10 ng/mL DEX was added), and LPS + DEX group (10 ng/mL DEX treated cells for 1 h, and then added 20 *μ*g/mL LPS) [11]. Later on, after incubation with the CCK-8 reagent (10 *μ*L) for 2 h, a microplate reader (PerkinElmer Envision) was used to measure the absorbance of RLE-6TN cells at a wavelength of 450 nm.

### 2.3. Cell Transfection

MiR-140-3p mimic, negative control (NC) mimic, three PD-L1 siRNA, and one siRNA negative control (NC) were synthesized by Sangon Biotech (Shanghai, China). The PD-L1 siRNA sequences were PD-L1 siRNA 1: 5′-CCUGUACGUGGUGGAGUAUTT-3′, PD-L1 siRNA 2: 5′-GCCGAAGUGAUCUGGACAATT-3′, and PD-L1 siRNA 3: 5′-GCGGCUUCGAAGAUAGAAATT-3′, and the NC siRNA sequences were: 5′-UUCUCCGAACGUGUCACGUTT-3′. RLE-6TN cells were transfected using Lipofectamine 2000 (Invitrogen, USA) for 24 hours according to the manufacturer's instructions.

### 2.4. Real-Time qPCR

Total RNA was extracted from cells using the TaKaRa MiniBEST Universal RNA Extraction Kit according to the manufacturer's procedure. Then, cDNA was synthesized by using miRNA first-strand cDNA Synthesis Kit (Vazyme) and Hifair®II 1st Strand cDNA Synthesis SuperMix (YEASEN, Shanghai). After that, Hieff® qPCR SYBR Green Master Mix (YEASEN, Shanghai) was used to perform the RT-qPCR reaction on an ABI QuantStudio™ 12 K Flex according to the manufacturer's procedure. The qPCR reaction conditions were as follows: 95°C for 5 mins followed by 40 cycles of 95°C for 10 s, 60°C for 30 s, and ﬁnally, 72°C for 20 s. miR-140-3p: F: 5′-GCGCGTACCACAGGGTAGAA-3′; R:5′-CCAGTGCAGGGTCCGAGGTATT-3′, PD-L1 : F: 5′-CTTGCCAAAGGACCAGCTTT-3′; R:5′-TGTCCAGATCACTTCGGCTT-3′, GAPDH : F: 5′-ACTCCCATTCTTCCACCTTTG-3′; R:5′-CCCTGTTGCTGTAGCCATATT-3′, U6 : F: 5′-GCTTCGGCAGCACATATACTAAAAT-3′; and R:5′-CGCTTCACGAATTTGCGTGTCAT-3′. The primers were obtained from Sangon Biotech (Shanghai, China). The levels of miR-140-3p were normalized to U6. The level of PD-L1 was normalized to GAPDH. The relative expression levels of genes were calculated using the 2^−ΔΔCt^ method.

### 2.5. Western Blot Analyses

Total proteins of cells were qualiﬁed by using a BCA protein assay kit (Thermo Fisher Scientiﬁc). The equal amounts of protein were separated by 12% SDS-PAGE and was transferred to a polyvinylidene fluoride membrane (HYBOND, CA). Membranes were blocked and then incubated overnight at 4°C with antibodies against PD-1/PD-L1, phosphorylated c-Jun N-terminal kinase (p-JNK), JNK, Bnip3, or GAPDH (1 : 1000, ABclonal). The membranes were incubated with secondary antibodies conjugated to horseradish peroxidase (1 : 5000; Beyotime Biotechnology, China) at room temperature for 2 hours. Protein bands were detected using an enhanced efficient chemiluminescence kit ECL (GE Healthcare, UK) and quantitated using Quantity One software (Bio-Rad Laboratories, UK). Band intensities were normalized to that of GAPDH.

### 2.6. Luciferase Reporter Assay

The wild-type (WT) and mutant PD-L1-3′UTR (MUT) plasmids were constructed by GenePharma (Shanghai, China). Cells were seeded in 24-well plates and grown for 24 h before transfection. Lipofectamine 2000 (Invitrogen, USA) was used to cotransfect miR-140-3p mimics or the mimic NC and wild-type or mutant PD-L1-3′UTR plasmids into cells. Transfected cells were harvested after 48 h and then analyzed using a Dual-Luciferase Reporter Assay System (Promega, USA).

### 2.7. Enzyme-Linked Immunosorbent Assay (ELISA)

The cytokine production of TNF-*α*, IL-6, and IL-8 in serum was assessed by the ELISA kit (R&D Systems, Minneapolis, MN) according to the manufacturer's recommendation.

### 2.8. Statistical Analysis

All data are expressed as mean ± standard deviation. Statistical analysis was carried out using SPSS 13.0. Mann–Whitney *U* tests were used for the comparison of means between two datasets. Tukey's multiple comparison test for comparisons among multiple groups. *p* < 0.05 was considered to indicate a statistically significant difference.

## 3. Results

### 3.1. DEX-Inhibited Apoptosis Induced by LPS

**Figure 1:**
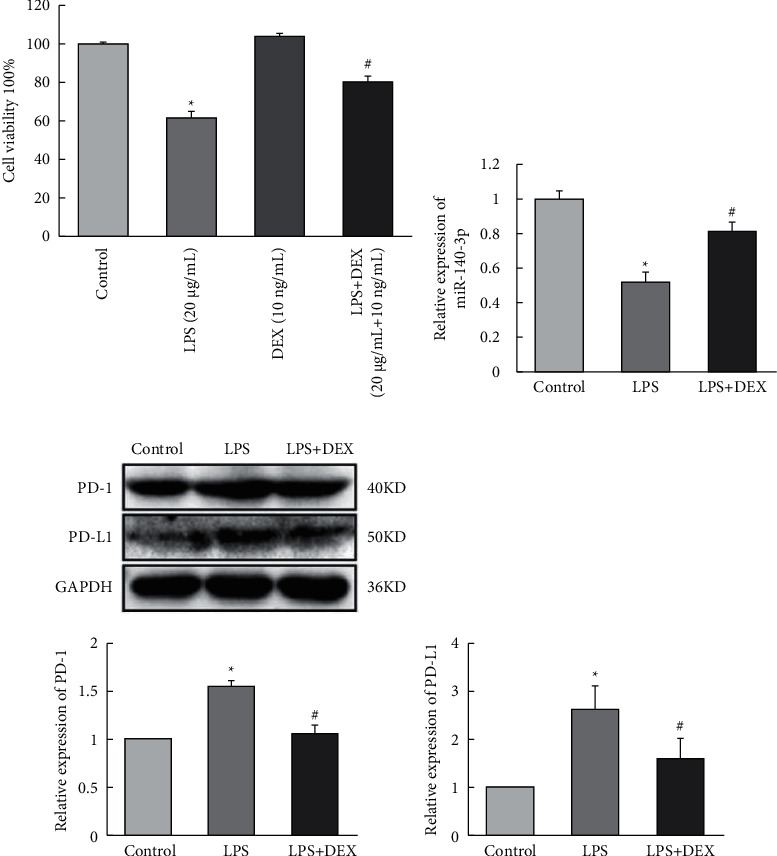
DEX protects alveolar epithelial cells from inflammation by promoting miR-140-3p level and suppressing PD-1/PD-L1 expression. (a) The survival rate of RLE-6TN cells induced by LPS and DEX. (b) The relative expression of miR-140-3p in RLE-6TN cells induced by LPS and DEX. The relative expression of PD-1/PD-L1 in RLE-6TN cells induced by LPS and DEX. Data are presented as means ± SD (*n* = 3; compared to control ^*∗*^*p* < 0.05, compared to LPS-induced ^#^*p* < 0.05).

We determined whether DEX exposure inhibited LPS-induced cell apoptosis. As seen in Figure 1(a), LPS exposure significantly decreased the survival rate of RLE-6TN cells to 61.35% when compared to control cells (*p* < 0.05). The survival rate increased to 80.13% for cells that were treated with DEX compared to cells that had been treated with LPS alone (*p* < 0.05).

### 3.2. DEX Increased miR-140-3p Expression That Had Been Downregulated by LPS Exposure

We determined whether miR-140-3p was important in the increased DEX-induced resistance to the inflammation that resulted from exposure to LPS. To investigate this possible association, we assessed the levels of miR-140-3p in the LPS- and DEX-treated (LPS + DEX) groups. We determined that the levels of miR-140-3p were decreased for RLE-6TN cells that had been exposed to LPS (Figure 1(b)). However, exposure to DEX reversed the downregulated miR-140-3p expression that LPS had induced. Therefore, DEX exposure appeared to reverse the LPS-induced inflammation of RLE-6TN cells by elevating expression levels of miR-140-3p.

### 3.3. DEX Inhibited PD-1/PD-L1 Induced by LPS

We examined effects of the LPS exposure on PD-1/PD-L1 expression. We also looked for an association between DEX resistance to inflammation in alveolar type II cells line with LPS exposure. Third, the PD-1/PD-L1 expression in cells treated with DEX was measured. We went on to measure PD-1/PD-L1 in the LPS- and DEX-treated (LPS + DEX) groups and determined that LPS exposure elevated overall PD-1/PD-L1 expression. However, after DEX treatment, we observed decreased amounts of PD-1/PD-L1 (Figure 1(c)). These observations indicated that the inflammatory response induced by LPS was accompanied by increased PD-1/PD-L1 levels, but DEX exposure reduced PD-1/PD-L1 levels, which then inhibited the inflammatory response.

### 3.4. DEX Inhibited PD-1/PD-L1 Levels by Partially Increasing miR-140-3p

**Figure 2:**
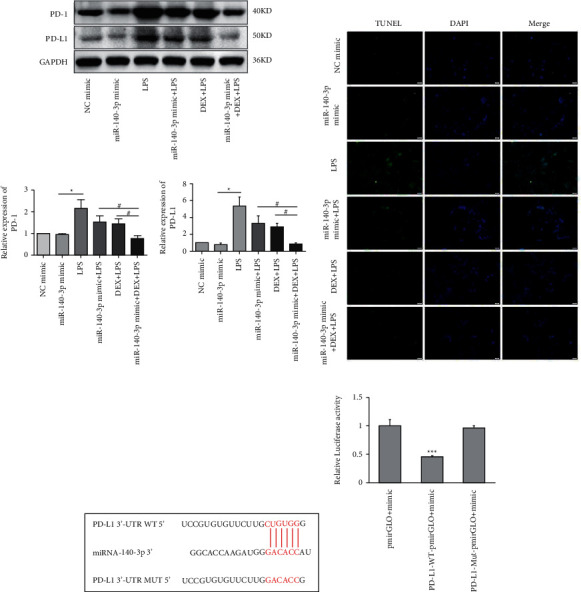
Effects of miR-140-3p overexpression on PD-1/PD-L1 expression and cell viability of RLE-6TN cells. (a) The relative expression of PD-1/PD-L1 in RLE-6TN cells transfected with miR-140-3p mimic. (b) The cell apoptosis of RLE-6TN cells transfected with miR-140-3p mimic detected by TUNEL. (c) Predicted complementary binding site of miR-140-3p in PD-L1 3′-UTR. (d) The relative luciferase activities were inhibited and transfected with the reporter vector WT PD-L1, not with the reporter vector Mut PD-L1. Data are presented as means ± SD (*n* = 3; compared to control ^*∗*^*p* < 0.05, compared to LPS-induced ^#^*p* < 0.05, compared to control ^*∗∗∗*^*p* < 0.01).

Due to the results seen above, we investigated whether the DEX inhibition of PD-1/PD-L1 was associated with changes in miR-140-3p concentrations. We increased miR-140-3p expression in RLE-6TN cells using a miR-140-3p mimic. We measured PD-1/PD-L1 levels in the LPS- and DEX-treated (LPS + DEX) groups. We confirmed that miR-140-3p levels were elevated, and DEX treatment reduced the PD-1/PD-L1 levels induced in LPS-exposed RLE-6TN cells (Figure 2(a)). Transfected with the miR-140-3p mimic decreased PD-L1 expression, but did not show obviously different. It may be that miR-140-3p mimic plays a partial effect on the expression of PD-L1, and there may be other regulatory genes affecting PD-L1 expression. Notably, combining DEX treatment with exposure to the miR-140-3p mimic inhibited PD-1/PD-L1 expression more effectively than DEX treatment alone or with exposure to the miR-140-3p mimic alone. These observations demonstrated miR-140-3p expression likely partially inhibited PD-L1 expression. Also, the DEX treatment of RLE-6TN cells increased miR-140-3p expression levels, which partially suppressed the increased levels of PD-L1 that had been induced by LPS exposure. DEX suppressed the expression of PD-L1 through the miR-140-3p-independent pathways. We used TUNEL staining to determine whether the inhibition of apoptosis by DEX treatment was related to the miR-140-3p concentrations (Figure 2(b)). Overexpression of miR-140-3p and treatment with DEX inhibited the apoptosis that was induced by exposure to LPS. Combining DEX treatment and exposure to the miR-140-3p mimic reduced apoptosis more effectively than treatment with only DEX or the miR-140-3p mimic.

### 3.5. miR-140-3p Targeted PD-L1 Directly, Which Partially Inhibited Expression of PD-L1 When Cells Were Exposed to LPS

Several studies using colorectal cancer cells have reported that miR-140-3p targets PD-L1 directly, resulting in the decreased expression of PD-L1. Therefore, we determined whether PD-L1 was directly targeted by miR-140-3p in ALI. As seen from Figure 2, PD-L1 was observed to be a predicted target gene. It was determined that a complementary miR-140-3p site was contained in the 3′-UTR end of PD-L1 (Figure 2(c)). Thus, an assay using a luciferase reporter was carried out to assess whether the 3′-UTR end of PD-L1 was directly targeted by miR-140-3p.  Figure 2(d) shows that miR-140-3p overexpression by 293T cells resulted in decreased luciferase activity that was exhibited by the wild-type PD-L1 3′-UTR reporter. On the other hand, the luciferase activity exhibited by the reporter was not apparently influenced by the presence of the miR-140-3p mimic when it included a mutated PD-L1 3′-UTR.

Interestingly, as Figure 2(a) documents, we noted that the PD-L1 levels were not decreased when the miR-140-3p mimic was transfected. However, in the LPS-exposed cells, miR-140-3p expression was observed to inhibit the increase in PD-L1 concentrations. We speculated that when LPS induction does not occur, the interactions of miR-140-3p and PD-L1 are balanced. However, even when miR-140-3p was overexpressed, it did not affect PD-L1 expression levels. Then, when LPS exposure did occur, cellular expression of PD-L1 increased. Thus, it appeared that PD-L1 was a direct target of miR-140-3p, which inhibited its expression.

### 3.6. The LPS-Induced JNK-Bnip3 Pathway Activation Was Inhibited by DEX through Promotion of miR-140-3p Expression

**Figure 3:**
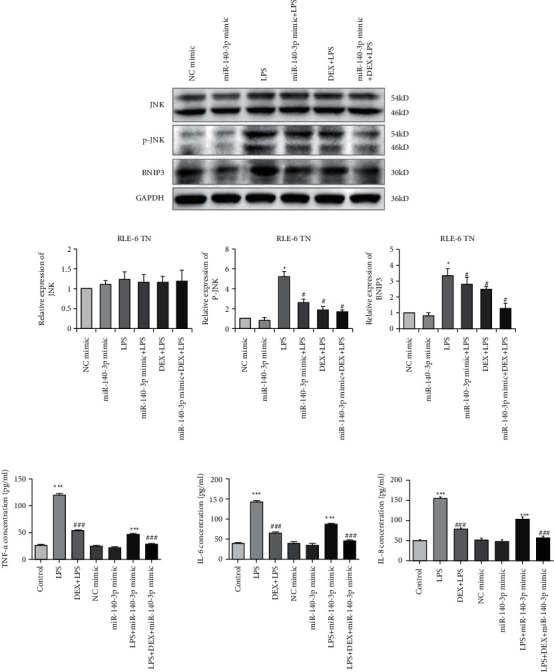
Effects of DEX and miR-140-3p overexpression on JNK-Bnip3 pathway. (a) The relative expression of JNK, p-JNK, and Bnip3. (b) The levels of TNF-*α*, IL-6, and IL-8 in RLE-6TN cells treated with DEX and miR-140-3p overexpressed. Data are presented as means ± SD (*n* = 3; compared to control ^*∗*^*p* < 0.05, compared to LPS-induced ^#^*p* < 0.05, compared to control ^*∗∗∗*^*p* < 0.01, compared to LPS-induced ^###^*p* < 0.01).

Several reports have indicated that injury caused by LPS-mediated inflammation is related to JNK-Bnip3 pathway activation. This pathway is associated with inhibition that prevents excessive autophagy and cell apoptosis that is induced by exposure to LPS. Therefore, we investigated whether this pathway played a role in the inhibition of autophagy that was mediated by the DEX treatment of alveolar type II cells. As shown in Figure 3(a), the phosphorylation of Bnip3, as well as JNK (p-JNK), was increased significantly in RLE-6TN cells (*p* < 0.05) after LPS treatment. Furthermore, miR-140-3p overexpression and DEX treatment attenuated these changes (*p* < 0.05). The combined exposure to the miR-140-3p mimic and DEX inhibited the levels of p-JNK and Bnip3 more effectively than treatment with only DEX or the miR-140-3p mimic. Thus, it appeared that DEX blocked the JNK-Bnip3 signaling pathway in RLE-6TN cells, which exhibited acute injury due to exposure to LPS by increasing miR-140-3p expression, which inhibited PD-1/PD-L1 expression.

### 3.7. DEX Exposure Increased miR-140-3p, Which Decreased Inflammatory Cytokine Levels

TNF-*α*, IL-6, and IL-8 have been reported to be pro-inflammatory mediators that are increased in several inflammatory diseases. To assess the anti-inflammatory effects of DEX or the miR-140-3p mimic, the levels of TNF-*α*, IL-6, and IL-8 were assessed in RLE-6TN cells.  Figure 3(b) shows that TNF-*α*, IL-6, and IL-8 were increased considerably in RLE-6TN cells that had been exposed to LPS (*p* < 0.05). However, cells that also were treated with DEX or transfected using a miR-140-3p mimic exhibited considerably reduced TNF-*α*, IL-6, and IL-8 levels in comparison with cells that had been treated with LPS alone (*p* < 0.05). Furthermore, the combined exposure to DEX and the miR-140-3p mimic suppressed the TNF-*α*, IL-6, and IL-8 levels more effectively than when cells were exposed to either DEX or a miR-140-3p mimic alone (*p* < 0.05). Thus, DEX appeared to have promoted miR-140-3p expression, which protected RLE-6TN cells from LPS-induced injury and inhibited the production of inflammatory cytokines.

### 3.8. Suppression of PD-L1 Expression Inactivated the JNK-Bnip3 Pathway

**Figure 4:**
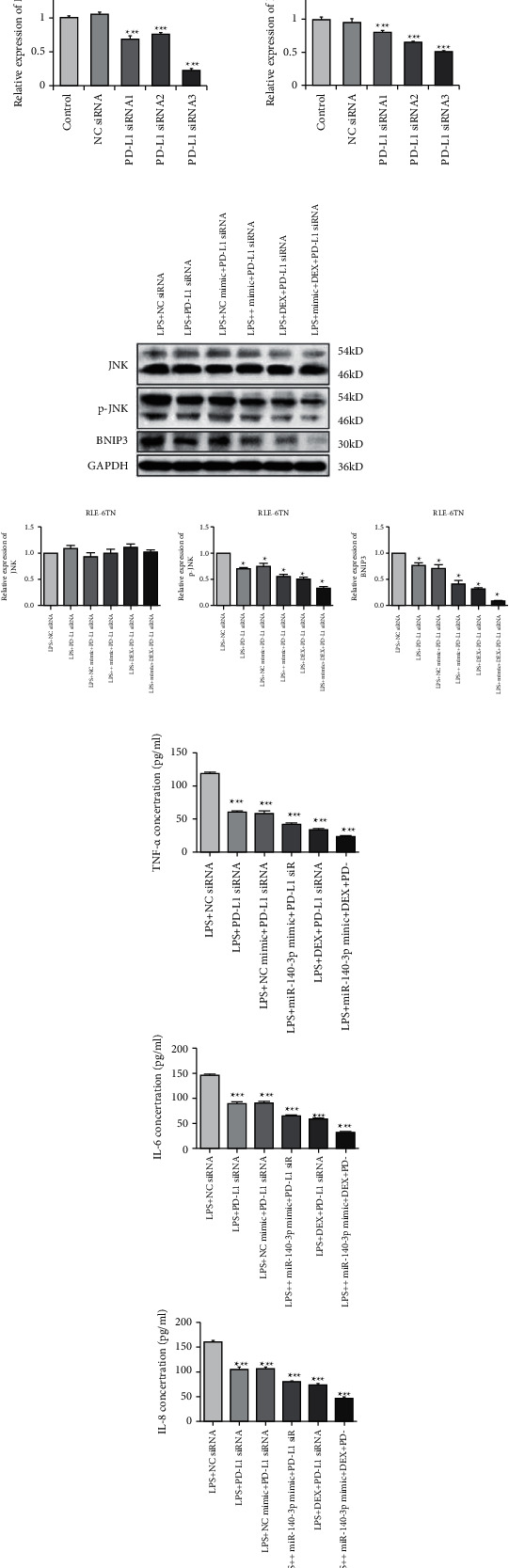
Effects of PD-L1 suppressing on JNK-Bnip3 pathway. (a) qPCR detected the relative expression of PD-L1 in RLE-6TN cells transfected with PD-L1 siRNAs. (b) Western blot detected the relative expression of PD-L1 in RLE-6TN cells transfected with PD-L1 siRNAs. (c) The relative expression of JNK, p-JNK, and Bnip3. (d) The levels of TNF-*α*, IL-6, and IL-8 in RLE-6TN cells. Data are presented as means ± SD (*n* = 3; ^*∗*^*p* < 0.05, ^*∗∗∗*^*p* < 0.01).

We also determined whether the suppression of PD-L1 could inhibit the JNK-Bnip3 signaling pathway. We constructed PD-L1 siRNA vectors and transfected them into RLE-6TN cells. As demonstrated in Figures 4(a) and 4(b), the concentrations of PD-L1 mRNA and protein decreased significantly in RLE-6TN cells that had been transfected with PD-L1 siRNAs. Moreover, PD-L1 expression was more effectively suppressed by PD-L1 siRNA-3.

We then evaluated the expression of Bnip3, JNK, and p-JNK in RLE-6TN cells transfected with PD-L1 siRNA-3. The data revealed that the inhibition of PD-L1 suppressed p-JNK and Bnip3 (Figure 4(c)). Furthermore, exposure to DEX and simultaneous miR-140-3p mimic transfection suppressed p-JNK as well as Bnip3 expression considerably more than when cells were transfected with PD-L1 siRNA alone. Thus, exposure to a combination of DEX, the miR-140-3p mimic, and PD-L1 siRNA inhibited p-JNK and Bnip3 expression better than any other single or combined treatment (*p* < 0.05).

We also assessed the amount of TNF-*α*, IL-6, and IL-8 in RLE-6TN cells.  Figure 4(d) shows that TNF-*α*, IL-6, and IL-8 expression were decreased substantially in PD-L1 siRNA-transfected RLE-6TN cells (*p* < 0.05). Moreover, TNF-*α*, IL-6, and IL-8 were clearly suppressed in cells that had been cotreated with DEX or the miR-140-3p mimic in comparison with cells exposed to PD-L1 siRNA alone (*p* < 0.05). Thus, the combined treatment with DEX, the miR-140-3p mimic, and PD-L1 siRNA inhibited TNF-*α*, IL-6, and IL-8 expression levels more effectively than any other single or combined treatment (*p* < 0.05).

These observations demonstrated that decreased PD-L1 expression resulting from exposure to DEX and miR-140-3p effectively inhibited the inflammatory response seen in LPS-induced lung injury and involved the inactivation of the JNK-Bnip3 pathway.

## 4. Discussion

ARDS is considered to be the most advanced type of ALI, and it is known to be caused by severe extrapulmonary influences as well as pathogenic factors. One central theory concerning the molecular mechanisms associated with ARDS/ALI is that an imbalance in the inflammatory response will intensify epithelial or endothelial injury. These responses produce increased permeability of the alveolar capillaries and subsequent fibrosis that results in the expression of ARDS.

DEX is a novel *α*-2 adrenoceptor agonist that produces anti-inflammatory responses in several diseases. Recent research has revealed that DEX is effective in improving pulmonary vascular permeability in animal studies and reduces inflammatory cytokine production in lung tissue, resulting in reduced inflammation [12, 13]. As Li et al. show that DEX can suppress lung injury in toxic shock rats by inhibiting inflammation and autophagy [14], DEX improves lung function by promoting inflammation resolution in patients undergoing totally thoracoscopic cardiac surgery [15]. It shows that the protective effect of DEX is closely related to the inhibition of inflammation. However, the mechanism by which DEX inhibits the inflammatory response has not been elucidated [16–18]. Studies have shown that DEX can regulate signaling pathways and genes associated with inflammation through interactions with several miRNAs [19]. For example, Bao et al. determined that DEX attenuated neuroinflammation associated with LPS-stimulated microglia cells by means of upregulating miR-340 [20]. DEX also attenuated apoptosis and inflammation in a liver cell line, L-02, caused by oxygen and glucose deprivation by means of the down-regulation of miR-711 [21]. We observed a significant reduction in miR-140-3p expression in LPS-induced ALI. However, this result can be rescued by DEX treatment that is provided before exposure to LPS. This information indicated that miR-140-3p could exhibit a regulatory function with respect to the protection from ALI provided by DEX.

Previous reports have revealed that PD-1 is commonly observed in a range of organs and tissues associated with the immune system. In addition, PD-L1 is extensively expressed as a primary ligand on both immune cells as well as nonimmune cells, such as alveolar epithelial cells [9]. Both PD-1 and PD-L1 have central roles concerning the development of indirect ARDS as well as indirect ALI. Xu et al. published that silencing endothelial cell expression of PD-L1 decreased indirect ALI development in a mouse model that was produced by inducing hemorrhagic shock with an ensuing septic challenge [22]. Joanne et.al suggested that PD-1/PD-L1 might exhibit novel roles with respect to functions associated with pulmonary vascular endothelial cells in the pathogenesis associated with indirect ARDS in mice [23]. In this set of experiments, we documented significantly increased PD-1 and PD-L1 levels in RLE-6TN cells in LPS-induced ALI. However, DEX was able to significantly inhibit the increased amounts of PD-1/PD-L1 observed with LPS induction. These results suggested that the protective effects provided by DEX might be related to decreased levels of PD-1/PD-L1.

A previous report documented that PD-1 was targeted by miR-140-3p directly and was able to suppress tumors in colorectal cancer [24]. Based on the results reported here, we suspected that miR-140-3p targeted PD-L1 directly and downregulated its expression to decrease the inflammation associated with ALI. The results showed that increased expression of miR-140-3p could reduce PD-L1 expression, although no significant difference was shown. However, combining DEX and the miR-140-3p mimic inhibited PD-L1 more effectively than when DEX or miR-140-3p mimic were applied individually. Moreover, the luciferase reporter assay provided verification that miR-140-3p exhibited a specific binding effect with PD-L1. It suggested that DEX suppressed the expression of PD-L1 through the miR-140-3p-independent pathways. Under normal conditions without LPS stimulation, PD-L1 levels were regulated both by miR-140-3p-dependent and miR-140-3p-independent pathways. However, when cells were exposed to LPS, PD-L1 was significantly increased. Then, miR-140-3p-targeted PD-L1, resulting in the partial inhibition of PD-L1 by the miR-140-3p-dependent pathway. Thus, only when acute lung injury results in an inflammatory response that leads to elevated PD-L1 expression will miR-140-3p exert a specific inhibitory effect on PD-L1 expression.

Several reports have suggested that the PD-L1 effects on various cell functions, including the immune response, involve activation of JNK. Kiriyama et al. indicated that nitric oxide-induced PD-L1 expression in A172 glioblastoma cells and the observed induction involved JNK activation [25]. Chen et al. demonstrated that exposure to nagilactone *E* increased PD-L1 expression levels in cells from lung cancers through the activation of the JNK-c-Jun axis [26].

JNK appears to have a critical role in inflammation and oxidative stress. JNK has been shown to be activated in lung injury, and the application of a JNK inhibitor can alleviate ALI [27, 28]. The inhibition of JNK phosphorylation is protective in LPS-induced ALI [29]. Bnip3 has been reported to be a proapoptotic protein that is mainly regulated by the JNK pathway. A recent study reported that Bnip3 is upregulated in inflammatory responses. Bnip3 has been shown to suppress inflammation as well as apoptosis induced by LPS exposure in chondrocytes through the promotion of autophagy [30]. The JNK-Bnip3 pathway has been reported to act as an upstream mediator of Bax upregulation, which can be activated by LPS to induce apoptosis of human olfactory ensheathing glial cells [31]. When the JNK-Bnip3 pathway was inhibited, Bax activation by LPS was eliminated, which resulted in attenuation of cell apoptosis.

Another study demonstrated that LPS exposure significantly increased p-JNK and Bnip3 expression. However, the p-JNK and Bnip3 expression were downregulated after DEX treatment, indicating that DEX could have a specific inhibitory effect with respect to the JNK-Bnip3 pathway. Moreover, the upregulation of miR-140-3p also inactivated the JNK-Bnip3 pathway, which was similar to the effects seen with DEX treatment. At the same time, the inhibition of PD-L1 achieved the same result with respect to the JNK-Bnip3 pathway. Therefore, we concluded that DEX inhibited PD-L1 expression by partially increasing miR-140-3p levels and inactivating the JNK-Bnip3 pathway in RLE-6TN cells. These changes in expression resulted in the inhibition of inflammation and alleviated ALI.

## 5. Conclusions

DEX was shown to inhibit the PD-L1 expression by partially promoting increased miR-140-3p levels in RLE-6TN cells. DEX also inactivated the JNK-Bnip3 pathway, resulting in inhibition of inflammation and alleviating alveolar type II cell injury. Therefore, DEX inhibited PD-L1 expression by partially increasing miR-140-3p levels and inactivating the JNK-Bnip3 pathway in RLE-6TN cells. These changes in expression resulted in the inhibition of inflammation and alleviated alveolar type II cell injury.

## Data Availability

All data used to support the findings of the study are included within the article.
